# 纵隔淋巴结清扫在肺癌诊疗中的共识与争议

**DOI:** 10.3779/j.issn.1009-3419.2018.03.10

**Published:** 2018-03-20

**Authors:** 兰军 张

**Affiliations:** 510060 广州，中山大学肿瘤防治中心胸外科 Department of Toracic Surgery, Sun Yat-sen University Cancer Center, Guangzhou 510060, China

**Keywords:** 肺肿瘤, 淋巴结清扫, 治疗, Lung neoplasms, Lymphadenectomy, Treatment

## Abstract

淋巴结转移是肺癌的重要转移途径，淋巴结清扫术已成为肺癌的标准术式，同时也决定着肺癌的分期、预后及治疗策略。在临床实践中肺癌淋巴结的清扫方式各有不同，从选择性的淋巴结采样到扩大的淋巴结清扫，目前各种清扫方式存在着很大的争议，本文就目前的纵隔淋巴结清扫方式的共识及争议进行综述，为今后开展多中心临床研究提供参考。

## Abstract (en)

Lymph node metastasis is an important route of metastasis of lung cancer. Lymphadenectomy has become the standard surgical procedure for lung cancer. Te way of intraoperative lymph node assessment also a?ects the prognosis and treatment strategy of lung cancer. In clinical practice, the way of intraoperative lymph node assessment ranges from selected lymph node biopsy to extended lymph node dissection. Te advantages and disadvantages of di?erent lymph node assessment are still controversial. In this article, the argument and consensus of lymphadenectomy on lung cancer operation are summarized.

非小细胞肺癌是目前世界上发病率及死亡率最高的恶性肿瘤之一。其总的生存率低，早期的肺癌5年生存率也只有60%左右^[1, 2]^。对于可切除的肺癌来说，手术是肺癌肿瘤治疗的最佳方案，而纵隔淋巴结清扫在肺癌手术中有着重要作用。现阶段对于肺癌淋巴结清扫方式的选择缺乏统一的共识，各方面存在诸多争议，本文就目前的纵隔淋巴结清扫方式的共识及争议进行综述。

## 1 肺癌淋巴结的分区及转移模式

区域的淋巴结状况对于肺癌患者精确的临床分期、治疗的选择及改善预后有着重要的参考和影响作用。Naruke^[3]^于1978年绘制了肺癌的淋巴结分区区域图，把区域淋巴结分成了14组。肺癌淋巴结的转移规律一般遵循着肺内淋巴结、肺门淋巴结、纵隔淋巴结的顺序，多数情况下肺癌淋巴结的转移按照这样的模式进行，但也有少数肺癌出现跳跃性转移、微转移(此种转移一般的病理切片无法发现，需采用免疫组织化学的检验方法)^[4-6]^及跳跃性微转移。

## 2 肺癌淋巴结的清扫方式

欧洲胸外科医师学会制定了肺癌淋巴结分期的指南，该指南对肺癌淋巴结清扫方式进行了定义及分类，分为：①选择性淋巴结活检，对手术中可疑转移的淋巴结进行采样活检，主要用于不能手术根治性切除的患者；②系统性淋巴结采样，系统性切取几枚预先选定的区域内的淋巴结，不做完全的清扫；③系统性淋巴结清扫，系统性清除解剖标志内包含淋巴结在内的所有纵隔组织，要求最少切除3站纵隔淋巴结，并且其中必须包括隆突下淋巴结，除纵隔淋巴结以外，肺门和肺内淋巴结必须一并切除；④肺叶特异性淋巴结清扫，根据原发肿瘤所在肺叶的不同，选择性清扫特定区域内包含淋巴结在内的纵隔组织；⑤扩大性淋巴结清扫，在常规系统性淋巴结清扫的前提下，扩大切除对侧纵隔淋巴结或颈部淋巴结^[7]^。

## 3 各指南对于淋巴结清扫的共识

美国国立综合癌症网络(National Comprehensive Cancer Network, NCCN)指南对于手术中淋巴结的清扫应常规做肺内及肺外淋巴结的切除，并要求至少完成三站以上的纵隔淋巴结取样或者完整切除。对于没有淋巴结转移的早期的肺癌其系统性的淋巴结采样也是容许的，对于每一站淋巴结应至少采样1个以上淋巴结，右侧的肺癌应采样右侧第2、4、7、8、9站淋巴结，左侧的肺癌应采样左侧第4、5、6、7、8、9站淋巴结。对于纵隔淋巴结有转移的肺癌患者应做系统性的同侧淋巴结清扫。对于亚肺叶切除的病人，在技术可行的情况下适当的选择性淋巴结取样也是推荐的^[8]^。

美国胸科医师协会(American College of Chest Physicians, ACCP)指南指出对于临床分期为Ⅰ期、Ⅱ期的肺癌患者系统性淋巴结清扫及采样都是推荐的病理分期方法，对于术前临床分期为Ⅰ期，术中淋巴结评估为纵隔淋巴结没有转移的肺癌患者，系统性淋巴结清扫并不能带来生存上的获益，因而推荐行选择性淋巴结的取样或者清扫。而对于临床分期为Ⅱ期的患者系统性淋巴结的清扫能带来更多生存上的获益^[9]^。

美国癌症联合会/国际抗癌联盟(American Joint Commitee on Cancer/Union for International Cancer Control, AJCC/UICC)组织对于肺癌病理分期的评估建议至少应评估6站以上淋巴结，每站至少一个淋巴结，建议清扫或采样3站以上N2淋巴结(其中包括第7组淋巴结)及3站以上N1淋巴结，对于总的淋巴结数目并没有清晰的要求，并推荐行系统性的淋巴结评估^[10, 11]^。

欧洲胸外科医师学会(European Society of Thoracic Surgeons, ESTS)指南推荐对所有肺癌患者行系统性淋巴结的取样或者清扫，特别是在N2的患者中，系统性的淋巴结评估会比选择性淋巴结评估带来更准确的分期及生存上的获益，在理想状态下建议行完整的淋巴结及周围组织的切除。总的要求肺癌手术中切除的淋巴结数目应大于6个。对于外周型的T1鳞癌患者肺叶特异性淋巴结清扫也是可以接受的，该指南还建议对于术中评估3站以上特定区域淋巴结是阴性的患者可不加做额外的淋巴结清扫，比如右中、上肺癌患者术中冰冻评估第2、4和7组是阴性，右中肺癌第4、7、8、9组是阴性，左上肺癌第5、6、7组淋巴结是阴性，左下肺癌第7、8、9组是阴性^[7]^。

## 4 纵隔淋巴结清扫的争议

对于纵隔淋巴结的清扫的争议集中于以下几个方面。

### 4.1 对于可切除的肺癌是行系统性的淋巴结清扫还是取样？

对比系统性淋巴结采样，系统性淋巴结清扫是否能够带来更准确的分期及生存的获益，而与之附带的是否也会增加手术的并发症：如喉返神经损伤、淋巴瘘、手术出血量的增多，住院时间的延长等，既往的研究^[12-14]^报道结论不一。一项大规模前瞻性的ACOSOG Z0030临床试验纳入了1, 023例随机患者，均为病理T1或T2/N0或N1，结果显示接受系统性淋巴结清扫的患者其手术并发症发生率和接受系统性淋巴结采样的患者没有差异，同样的，两组的局部及远处的复发率及生存率也没有差异，但系统性淋巴结清扫能够给患者带来更准确的分期并提供机会性术后辅助治疗^[15, 16]^。

### 4.2 对于早期肺癌是选择系统性淋巴结清扫还是选择性淋巴结清扫？

1999年，Asamura等^[17, 18]^报道了不同位置的肺癌其淋巴结转移特点，上肺叶肺癌容易出现上纵隔淋巴结的转移而单站的隆突下淋巴结转移少见，下肺叶肺癌容易出现下纵隔淋巴结转移，一般从隆突下淋巴结转移至上纵隔淋巴结。选择性淋巴结清扫(肺特异性淋巴结清扫)指的是下叶肺癌只清扫隆突下及下纵隔淋巴结，上叶肺癌只清扫上纵隔淋巴结。对于早期肺癌术中没有发现N2转移的患者，Okada等^[17, 19-21]^的研究结果均没发现选择性淋巴结清扫和系统性淋巴结清扫组两者生存上及复发率上的差异，在有些报道结果显示选择性淋巴结清扫在术后并发症发生率上有优势。但选择性淋巴结清扫的缺点是可能遗漏很少部分隐匿性的N2患者，这也是选择性淋巴结清扫带来的问题，还需进一步的前瞻性临床试验进行验证。

### 4.3 对于接受亚肺叶切除的肺癌患者是否需要行淋巴结清扫？

肺部磨玻璃结节其淋巴结转移概率低(尤其对于不典型腺瘤样变、原位腺癌、微浸润腺癌的病理类型)^[22]^。有些研究者甚至提出对于磨玻璃结节患者可以不用接受标准的肺叶切除及淋巴结清扫，但相关研究数据还较少，部分对于磨玻璃结节的手术方式选择的研究仍处于临床试验阶段，Moon等^[23]^的一项回顾性研究显示对于以磨玻璃为主的小于3 cm的结节其淋巴结转移率非常低，不进行淋巴结清扫的患者与进行淋巴结清扫或采样的患者其术后复发率无明显差异，但因其是一项回顾性研究，其结果仍需要前瞻性临床试验证实。

### 4.4 纵隔淋巴结清扫数目的界定

既往的多数研究发现淋巴结清扫的个数与肺癌的预后相关，结果显示清扫越多的淋巴结其生存预后较好，部分文章建议清扫的淋巴结个数在8个-16个之间不等，但各个研究的数据不一。对于准确的淋巴结清扫个数还没有统一的共识^[22-26]^。Riquet等^[27]^的研究数据建议清扫淋巴结个数在4个-10个以上，在不同分期的肺癌患者中其清扫淋巴结个数对预后的影响也有差异，越晚期的患者，淋巴结清扫个数达到一定阈值后其对预后的影响越小。故清扫淋巴结个数对预后是否有直接作用仍有争议，有研究者认为清扫淋巴结数目的增多可以提高分期的准确性及指导辅助治疗，并不对预后产生影响。总的来说纵隔淋巴结清扫数目也间接反映了肺癌淋巴结状态评估的重要性，也是对于系统性淋巴结清扫的间接肯定^[28]^。

### 4.5 对局部晚期肺癌是否需要扩大清扫？

对于局部晚期肺癌N2、Ⅲa期的患者，NCCN指南并没有建议行淋巴结扩大切除，只是对手术切缘(阴性)及手术是否完整切除(R0切除)做了要求，对于IIIa期的患者手术治疗的地位仍未得到巩固，对于手术中是否行扩大性淋巴结清扫仍存有较大争议^[8]^。以往扩大性淋巴结清扫因其手术创伤大，切除范围较大，较少被外科医生接受，但随着经颈纵隔镜及电视纵隔镜的发展，使得双侧纵隔淋巴结清扫成为一种创伤较小的手术方式^[29-31]^。Kuzdzal等^[32, 33]^的研究发现经颈纵隔淋巴结扩大清扫术可以准确地评估淋巴结转移状态，其手术并发症及创伤小，而且研究发现左下肺癌出现右侧上纵隔淋巴结转移的患者，其扩大性的淋巴结清扫能给其带来生存上的获益。经颈纵隔淋巴结扩大清扫可以为患者准确地行病理分期，从而指导新辅助治疗，并同时为患者尽量达到完整切除的治疗目的，使得局部晚期患者得到生存上的获益^[30]^。但扩大性淋巴结清扫因仍缺乏大量的研究数据的支持，现未被各项指南推荐。欧洲关于经颈双侧纵隔淋巴结扩大清扫的临床试验仍正在进行中^[34]^。

## 5 总结

对于可切除的肺癌，系统性淋巴结的评估是必要的，它是进行准确分期和指导治疗的重要手段，可选择系统性淋巴结清扫或者采样。淋巴结清扫方式的选择应根据肺癌的病理类型、分期情况及患者的基本身体情况来选择，比如选择性淋巴结清扫可在早期肺癌的患者中进行。现阶段不同地区及不同的指南对于淋巴结清扫方式的选择各有差异，完全规范的纵隔淋巴结清扫仍是主流，未来淋巴结清扫方式的选择应有大规模的临床试验进行验证。
